# Rule out of acute aortic dissection with plasma matrix metalloproteinase 8 in the emergency department

**DOI:** 10.1186/cc12536

**Published:** 2013-02-25

**Authors:** Francesca Giachino, Marilena Loiacono, Manuela Lucchiari, Maria Manzo, Stefania Battista, Elisa Saglio, Enrico Lupia, Corrado Moiraghi, Emilio Hirsch, Giulio Mengozzi, Fulvio Morello

**Affiliations:** 1 S.C. Medicina d'Urgenza, Molinette Hospital, A.O. Città della Salute e della Scienza di Torino, Corso Bramante, 88, 10126 Torino, Italy; 2 S.C. Chimica Clinica, Molinette Hospital, A.O. Città della Salute e della Scienza di Torino, Corso Bramante, 88, 10126 Torino, Italy; 3 Molecular Biotechnology Center, Università degli Studi di Torino, Via Nizza, 52, 10126 Torino, Italy

## Abstract

**Introduction:**

Matrix metalloproteinases (MMPs) are involved in aortic pathophysiology. Preliminary studies have detected increased plasma levels of MMP8 and MMP9 in patients with acute aortic dissection (AAD). However, the performance of plasma MMP8 and MMP9 for the diagnosis of AAD in the emergency department is at present unknown.

**Methods:**

The levels of MMP8 and MMP9 were measured by ELISA on plasma samples obtained from 126 consecutive patients evaluated in the emergency department for suspected AAD. All patients were subjected to urgent computed tomography (CT) scan for final diagnosis.

**Results:**

In the study cohort (N = 126), AAD was diagnosed in 52 patients and ruled out in 74 patients. Median plasma MMP8 levels were 36.4 (interquartile range 24.8 to 69.3) ng/ml in patients with AAD and 13.2 (8.1 to 31.8) ng/ml in patients receiving an alternative final diagnosis (*P *<0.0001). Median plasma MMP9 levels were 169.2 (93.0 to 261.8) ng/ml in patients with AAD and 80.5 (41.8 to 140.6) ng/ml in patients receiving an alternative final diagnosis (*P *= 0.001). The area under the curve (AUC) on receiver-operating characteristic (ROC) analysis of MMP8 and MMP9 for the diagnosis of AAD was respectively 0.75 and 0.70, as compared to 0.87 of D-dimer. At the cutoff of 3.6 ng/ml, plasma MMP8 had a sensitivity of 100.0% (95% CI, 93.2% to 100.0%) and a specificity of 9.5% (95% CI, 3.9% to 18.5%) and ruled out AAD in 5.6% of patients. Combination of plasma MMP8 with D-dimer increased the AUC on ROC analysis to 0.89. Presence of MMP8 <11.0 ng/ml and D-dimer <1.0 or <2.0 µg/ml provided a negative predictive value of 100% and ruled out AAD in 13.6% and 21.4% of patients respectively.

**Conclusions:**

Low levels of plasma MMP8 can rule out AAD in a minority of patients. Combination of plasma MMP8 and D-dimer at individually suboptimal cutoffs could safely rule out AAD in a substantial proportion of patients evaluated in the emergency department.

## Introduction

The prompt identification of acute aortic dissection (AAD) in the emergency department (ED) is paramount to reduce morbidity and mortality in affected patients, as diagnostic delays lead to inappropriate clinical management and defer life-saving treatments such as cardiothoracic surgery and/or endovascular repair [1,2]. However, the diagnosis of AAD in the ED is highly challenging, because AAD is rare (2-4 cases per 100,000 individuals per year) and clinically heterogeneous at presentation [3-5]. Currently, the diagnostic approach to suspected AAD relies on imaging techniques such as computed tomography (CT) scan and transesophageal echocardiography, which nonetheless require thorough pretest clinical selection and may not be immediately available in all EDs [2]. Therefore, identification of blood markers supporting or refusing the diagnosis of AAD would provide a major breakthrough. D-dimer, a well-established marker of vascular thrombosis, has shown high sensitivity but low specificity for the diagnosis of AAD [6,7]. Ideal diagnostic markers of AAD would be components of the aortic wall released into the bloodstream upon acute aortic damage, similar to circulating troponin for acute myocardial damage. On these grounds, different aortic proteins have been examined, such as smooth muscle myosin heavy chain, soluble elastin fragments and calponin [8-10]. However, none of them has been introduced into clinical practice so far.

Matrix metalloproteinases (MMPs) constitute a large family of calcium and zinc-dependent endopeptidases that degrade the extracellular matrix [11]. MMPs are key molecular mediators of aortic disease and contribute to the landmark feature of extracellular matrix fragmentation underlying AAD [12]. In particular, several lines of evidence have shown that MMP9 (collagenase type IV or gelatinase B) is activated in human aortic aneurysms [13-16]. Instead, less is known about MMP8 (neutrophil collagenase I) in aortic disease.

Several studies have demonstrated an association between aortic pathology and the circulating levels of MMPs [17]. The plasma levels of MMP8 and MMP9 have also been shown to increase in AAD compared to healthy controls and to selected patients with acute coronary syndromes [18-21]. However, the utility of circulating MMP8 or MMP9 for the diagnosis of AAD in the ED depends on their actual plasma levels in the far broader spectrum of patients with clinically suspected AAD.

To evaluate the diagnostic performance of plasma MMP8 and MMP9, their levels were evaluated in a prospective cohort of patients managed in the ED for suspected AAD. The diagnostic performance of MMP8 and MMP9 was also compared to that of D-dimer, the only routinely available circulating marker relevant to AAD.

## Materials and methods

### Study population

The present study was approved by the local Ethics Committee (Comitato Etico Interaziendale A.O.U. San Giovanni Battista di Torino e A.O. C.T.O. Maria Adelaide di Torino) and was performed in accordance with the Declaration of Helsinki (1964). All patients included in the study provided their informed consent (i) to enrolment and (ii) to publication of scientific results. The plasma levels of MMP8 and MMP9 were assayed in consecutive patients evaluated for suspected AAD in the ED of the Molinette Hospital of Turin, Italy, for a total of 26 months starting from June 2010. Blood samples for MMP dosage were obtained in the ED at the time of routine clinical evaluation. All patients included in the study were evaluated within 24 hours after symptom onset.

Patients were eligible to the study if all the following criteria were met: (i) clinical presentation suggestive of AAD (chest pain, abdominal pain, back pain, syncope or symptoms of perfusion deficit), as indicated by the 2010 guidelines of the American Heart Association [22]; (ii) order of an urgent chest and abdomen CT with contrast by the caring physician to expressly rule out AAD; (iii) informed consent of the participant. To confirm appropriateness of enrolment, cases were subsequently re-evaluated by independent reviewers based on clinical charts and written requests for urgent CT originally filled out by the caring physician. Reviewers were blinded to the results of CT scan, blood tests and final diagnosis. Final alternative diagnoses were based on patient dismissal for hospitalized patients and on ED dismissal for outpatients.

### Matrix metalloproteinases

Blood samples were obtained from all patients in EDTA-containing tubes during their routine medical evaluation in the ED, when the other blood samples for routine diagnostic tests were drawn, and immediately sent to the laboratory. Plasma was obtained by centrifugation at 4,000 rpm for 5 minutes and stored at -80ºC until further processing. Plasma levels of MMP8 and MMP9 were measured by ELISA (Amersham™; GE Healthcare, Chalfont St Giles, UK). The assay is based on a two-site ELISA sandwich format using two antibodies (for capture and detection) directed against different epitopes of MMPs. The precision analysis for measurement of MMP8 and MMP9 yielded coefficients of variation (CV%) of 2.3% and 5.5%, respectively for within assay tests, and of 2.5% and 9.8% respectively for between-assay tests.

### Biochemical analyses

Creatinine, troponin T, lactate dehydrogenase (LDH) and C-reactive protein (CRP) were measured with the automated Cobas C8000 chemistry analyzers (Roche Diagnostics GmbH, Mannheim, Germany). D-dimer levels were measured with the automated STA LIATEST^™ ^D-DI, based on latex agglutination (Diagnostica Stago for Roche Diagnostics GmbH, Mannheim, Germany).

### Statistical analysis

Statistical analysis was performed with GraphPad Prism Software version 5.0 (GraphPad Software, San Diego, CA, USA). Data are presented as median and interquartile range (IQR), as the distribution of all biochemical variables (including MMP8 and MMP9) in the study population was not normal. Statistical tests used in the study were nonparametric Mann-Whitney *U *test, nonparametric Dunn's test for multiple comparisons, Fisher's exact test and nonparametric Spearman correlation. Statistical comparison of different ROC curves was performed as previously described [23]. *P *values lower than 0.05 were considered as statistically significant.

## Results

### Patient cohort

In the study period, 186 patients managed in the ED for suspected AAD were eligible for the study. The number of enrolled patients was 126 (67.7%). In these individuals, chief complaints were chest pain (60%), abdominal pain (17%), back pain (30%), syncope (5%) and other symptoms (17 %), in line with previous reports [3]. In this cohort, AAD was finally diagnosed in 52 patients (41.3%), including 32 cases of Stanford type A and 20 cases of Stanford type B AAD. AAD was initially suspected but finally ruled out by CT scan in 74 patients (58.7%). In these individuals, alternative final diagnoses included aortic aneurysm without signs of dissection (*n *= 16), inflammatory disease (*n *= 6), acute coronary syndrome (*n *= 5), other diagnoses (*n *= 8) and uncertain diagnoses (*n *= 39). The demographic and clinical characteristics of enrolled individuals are presented in Table 1.

**Table 1:** Demographic and nosological characteristics of enrolled patients.

	AAD	Alternative diagnosis	*P*
N	52 (41.3%)	74 (58.7%)	

	Stanford	Alternative diagnoses:	
	classification:	- Uncomplicated aortic aneurysm (16)	
	- type A AAD (32)	- Inflammatory disease (6)	
	- type B AAD (20)	- Pneumonia (3), pericarditis (1), pancreatitis (1), urinary tract infection (1)	
		- Acute coronary syndrome (5)	
		- Other diagnoses (8)	
		- Uncertain diagnoses (39)	

Gender (M/F)	40/12	49/25	0.24

Age (years)	66.3 ± 1.9	64.0 ± 1.7	0.39

Hypertension	31 (59.6%)	44 (59.5%)	1.00

Diabetes	1 (1.9 %)	11 (14.9%)	0.02

Age is reported as mean ± standard deviation. *P *values are calculated with Fisher's exact test. AAD, acute aortic dissection.

### Plasma levels of MMP8 and MMP9

The routine biochemical profile of patients with AAD and of patients receiving an alternative final diagnosis is presented in Table 2. Median D-dimer levels were 7.16 µg/ml (2.81 to 18.70) in patients with AAD and 1.34 µg/ml (0.35 to 2.31) in patients receiving an alternative final diagnosis (*P *<0.0001), in line with previous reports [6,7]. Also LDH was significantly higher in AAD compared to controls (*P *= 0.02). No significant differences were observed in the other biochemical variables.

**Table 2:** Biochemical profile of enrolled patients.

	AAD	Alternative diagnosis	*P*
Creatinine (mg/dl)	1.02 (0.86-1.32) [N = 48]	0.99 (0.77-1.31) [N = 71]	0.21
LDH (IU/l)	415 (370-651) [N = 23]	371 (329-424) [N = 42]	0.02
Troponin-T (μg/dl)	0.010 (0.006-0.029) [N = 45]	0.010 (0.008-0.013) [N = 67]	0.69
CRP (mg/dl)	4.4 (1.6-34.3) [N = 40]	5.2 (0.9-17.5) [N = 63]	0.36
D-dimer (µg/ml)	7.16 (2.81-18.70) [N = 42]	1.34 (0.35-2.31) [N = 61]	0.0001

Values are presented as median and interquartile range (IQR). N represents the sample number for each biochemical value. *P *values are calculated with nonparametric Mann-Whitney *U *test. AAD, acute aortic dissection; CRP, C-reactive protein; LDH, lactate dehydrogenase.

**Figure 1:**
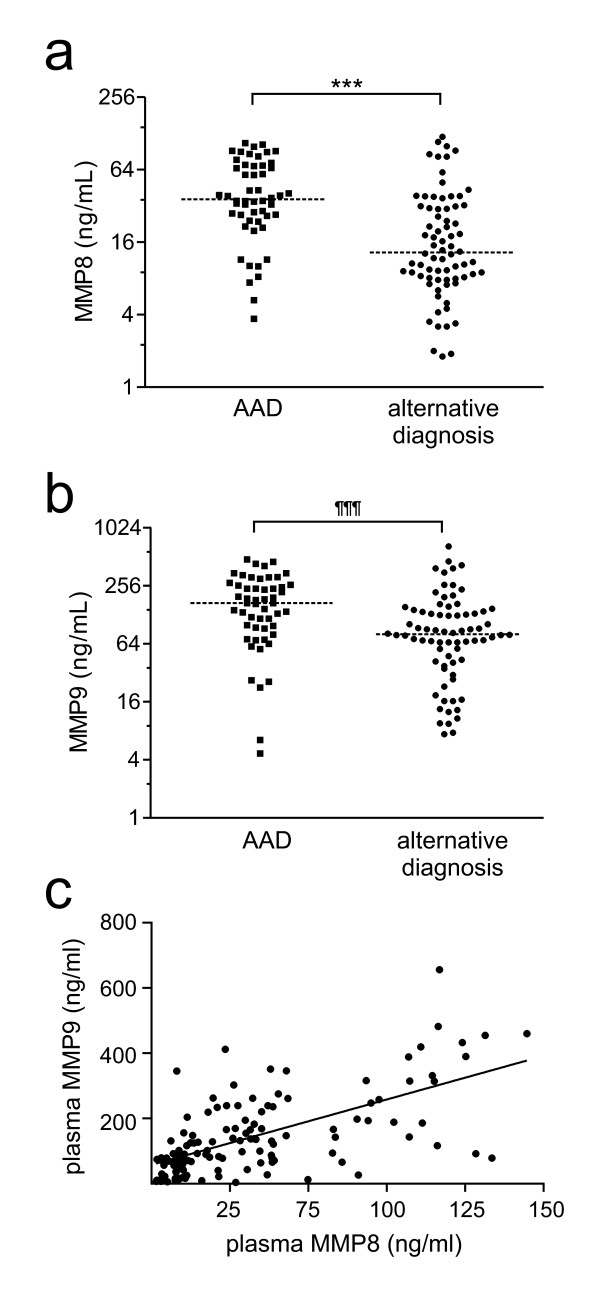
**Plasma levels (ng/ml) of MMP8 (a) and MMP9 (b) in patients with acute aortic dissection (AAD) and in patients receiving a final alternative diagnosis**. Y-axis: log_2 _scale. ***P <0.0001, ^¶¶¶^*P *= 0.0001 by nonparametric Mann-Whitney *U *test. **(c) **Correlation of plasma MMP8 and plasma MMP9 in enrolled patients. MMP, matrix metalloproteinase.

Median MMP8 levels were 36.4 ng/ml (24.8 to 69.3) in patients with AAD and 13.2 ng/ml (8.1 to 31.8) in patients receiving a final alternative diagnosis (*P *<0.0001, Figure 1a). MMP8 levels were 39.9 ng/ml (26.8 to 72.3) in patients with Stanford type A AAD and 34.2 ng/ml (14.7 to 64.2) in patients with Stanford type B AAD (*P *= 0.32). MMP8 levels of control patients according to alternative final diagnoses are reported in Table 3. In patients receiving an alternative final diagnosis, the highest levels of plasma MMP8 were observed in individuals affected by an inflammatory disease. Of note, plasma MMP8 was not increased in patients with uncomplicated aortic aneurysm, suggesting that aortic dilation *per se *is not sufficient to significantly increase the circulating levels of MMP8 in this patient population.

**Table 3:** Plasma levels of MMP8 and MMP9 in acute aortic dissection versus alternative diagnoses.

	N	MMP8 (ng/ml)	MMP9 (ng/ml)
Acute aortic dissection	52	36.4 (24.8-69.3)^a,f^	169.2 (93.0-261.8)^e,g^
Uncomplicated aortic aneurysm	16	10.7 (4.6-33.9)^a^	54.4 (20.9-96.6)^e,b^
Inflammatory disease	6	60.8 (31.6-105.5)^d^	294.0 (125.8-407.6)^b,c^
Acute coronary syndrome	5	12.90 (8.1-29.0)	92.4 (58.1-152.5)
Other diagnoses	8	17.6 (12.9-69.3)	137.1 (63.3-340.5)
Uncertain diagnoses	39	10.5 (7.3-22.9)^d,f^	78.8 (35.4-127.6)^c,g^

Values are presented as median and interquartile range (IQR). ^a,b,c^*P *<0.05; ^d,e^*P *<0.01; ^f,g^*P *<0.001 by nonparametric Dunn's multiple comparison test. MMP, matrix metalloproteinase.

Median MMP9 levels were 169.2 ng/ml (93.0 to 261.8) in patients with AAD and 80.5 ng/ml (41.8 to 140.6) in patients receiving a final alternative diagnosis (*P *= 0.0001, Figure 1b). Median MMP9 levels were 204.9 ng/ml (104.1 to 296.3) in patients with Stanford type A dissection and 126.6 ng/ml (70.91 to 197.0) in patients with type B dissection (*P *= 0.13). Plasma MMP9 levels of control patients according to alternative diagnoses are reported in Table 3. In patients receiving an alternative final diagnosis, the highest levels of plasma MMP9 were also observed in individuals affected by an inflammatory disease. Of note, plasma MMP9 was not increased in patients with uncomplicated aortic aneurysm, suggesting that aortic dilation *per se *is not sufficient to significantly increase circulating levels of MMP9 in this patient population.

### Correlation of plasma MMPs with CRP and D-dimer

Plasma levels of MMP8 and MMP9 were highly correlated (Figure 1c, Spearman r = 0.63, *P *<0.0001). Neither plasma MMP8 nor plasma MMP9 correlated with creatinine. Plasma MMP8 presented a slight albeit significant correlation with CRP (Spearman r = 0.22, *P *= 0.022) and a stronger correlation with D-dimer (Spearman r = 0.32, *P *<0.001). Also MMP9 correlated with both CRP (Spearman r = 0.29, *P *= 0.003) and D-dimer (Spearman r = 0.27, *P *= 0.005).

### Receiver-operating characteristics curve analysis of plasma MMP8 and MMP9

**Figure 2:**
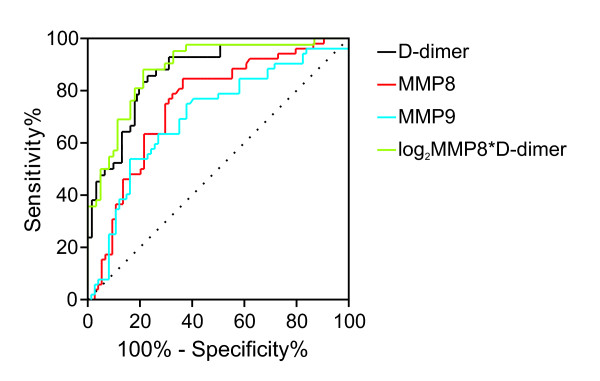
Receiver-operating characteristics (ROC) curve of plasma MMP8, plasma MMP9, D-dimer and combination of MMP8 and D-dimer (log_2_MMP8*D-dimer) for patients with acute aortic dissection versus subjects receiving an alternative final diagnosis. MMP, matrix metalloproteinase.

The receiver-operating characteristics (ROC) curves of MMP8, MMP9 and D-dimer for the diagnosis of AAD are presented in Figure 2. For D-dimer, the AUC on ROC analysis for patients with AAD was 0.87 (95% CI, 0.80 to 0.94) versus all controls, in line with previous reports [7]. Using a cutoff of 0.50 µg/ml, D-dimer showed a sensitivity of 97.6% (95% CI, 87.4% to 99.9%) and a specificity of 32.8% (95% CI, 21.3% to 46.0%). In the study cohort, D-dimer levels were <0.50 µg/ml in 1/42 (2.4%) patients with AAD (false negatives) and in 20/61 (32.8%) patients without AAD (true negatives).

For MMP8, the AUC on ROC analysis for patients with AAD was 0.75 (95% CI, 0.66 to 0.84) versus all controls, 0.75 (95% CI, 0.59 to 0.91) versus aortic aneurysm without dissection, 0.67 (95% CI, 0.42 to 0.91) versus inflammatory disease, 0.82 (95% CI, 0.69 to 0.95) versus acute coronary syndrome, 0.66 (95% CI, 0.44 to 0.88) versus other diagnoses and 0.82 (95% CI, 0.73 to 0.91) versus uncertain diagnoses. Using a cutoff of 3.6 ng/ml, plasma MMP8 had a sensitivity of 100.0% (95% CI, 93.2% to 100.0%) and a specificity of 9.5% (95% CI, 3.9% to 18.5%). In the study cohort, plasma MMP8 levels were <3.6 ng/ml in 0/52 patients (0%) with AAD (false negatives) and in 7/74 (9.5%) patients without AAD (true negatives).

For MMP9, the AUC on ROC analysis for patients with AAD was 0.70 (95% CI, 0.61 to 0.80) versus all controls, 0.77 (95% CI, 0.63 to 0.91) versus aortic aneurysm without signs of dissection, 0.69 (95% CI, 0.45 to 0.93) versus inflammatory disease, 0.73 (95% CI, 0.56 to 0.89) versus acute coronary syndrome, 0.54 (95% CI, 0.31 to 0.78) versus other diagnoses and 0.76 (95% CI, 0.66 to 0.86) versus uncertain diagnoses. Using a cutoff of 20.0 ng/ml, plasma MMP9 had a sensitivity of 96.2% (95% CI, 86.8% to 99.5%) and a specificity of 16.2% (95% CI, 8.7% to 26.6%). In the study cohort, plasma MMP9 levels were <20.0 ng/ml in 2/52 (3.8%) patients with AAD (false negatives) and in 12/74 (16.2%) patients without AAD (true negatives).

### Combination of plasma MMP8 with D-dimer

Next, we tested the hypothesis that a combined use of plasma MMP8 and D-dimer may: (i) increase the diagnostic accuracy of MMP8 alone, and (ii) safely rule out AAD in a clinically significant proportion of patients. Indeed, calculation of the AUC on ROC analysis demonstrated better performance of a combination of MMP8 and D-dimer than plasma MMP8 alone (*P *<0.05, Figure 2 and Table 4). Table 5 reports the negative predictive values (NPV) obtained by combination of three different diagnostic cutoffs for MMP8 and D-dimer. Of note, the NPV of plasma MMP8 at the cutoff of 11.0 ng/ml increased from 84.2% when used alone to 100% when used in association with any proposed D-dimer cutoff (0.5, 1.0 or 2.0 µg/ml). Furthermore, combination of MMP8 <11.0 ng/ml with any D-dimer cutoff identified up to 22 true negatives in the study cohort and was not associated with false negatives, as compared to 6 false negatives with MMP8 <11.0 ng/ml alone. These findings indicate that a combined use of plasma MMP8 and D-dimer represents a promising strategy to safely rule out AAD in a significant proportion of patients in the ED.

**Table 4:** ROC analysis for plasma MMP8, D-dimer and their combination.

	AUC (95% CI)	Cutoff	Sensitivity % (95% CI)	Specificity % (95% CI)
MMP8	0.75^a,b,c^ (0.66-0.84)	>3.6 ng/ml	100% (93.2%-100.0%)	9.5% (3.9%-18.5%)

DD	0.87^a^ (0.80-0.94)	>0.50 µg/ml	97.6% (87.4%-99.9%)	32.8% (21.3%-46.0%)

DD*MMP8	0.87^b^ (0.80-0.94)	>2.44	100% (91.6%-100.0%)	16.4% (8.2%- 28.1%)

DD*log_2_(MMP8)	0.89^c^ (0.82-0.95)	>0.77	100% (91.6%-100.0%)	13.1% (5.8%- 24.2%)

^a^*P *= 0.048, ^b^*P *= 0.034, ^c^*P *= 0.018. AUC, area under the curve; DD, D-dimer; MMP, matrix metalloproteinase; ROC, receiver-operating characteristics.

**Table 5:** Negative predictive value of plasma MMP8 in combination with D-dimer.

NPV		D-dimer (µg/ml)			
		
	AND	Any value	<0.5	<1.0	<2.0
	**<3.6**	100% TN 7, FN 0	100% TN 1, FN 0	100% TN 1, FN 0	100% TN 3, FN 0
	
**MMP8 (ng/ml)**	**<11.0**	84.2% TN 32, FN 6	100% TN 12, FN 0	100% TN 14, FN 0	100% TN 22, FN 0
	
	**<30.0**	73.2% TN 52, FN 19	94.1% TN 16, FN 1	95.5% TN 21, FN 1	93.9% TN 31, FN 2
	
	**Any value**		95.2% TN 20, FN 1	96.6% TN 28, FN 1	93.3% TN 42, FN 3

Three representative cutoffs are presented for each biomarker. NPV, negative predictive value (%); TN, true negatives; FN, false negatives; MMP, matrix metalloproteinase.

## Discussion

The present study is, to the best of our knowledge, the first to evaluate the levels of plasma MMP8 and MMP9 in a prospective cohort of patients managed in the ED for suspected AAD, and thus to directly estimate the actual diagnostic value of these markers. Previous case-control studies have reported increased plasma levels of MMP8 and MMP9 in AAD [18,20]. These studies had a retrospective design and focused on selected groups as controls, such as normal individuals and patients with acute coronary syndrome. However, the differential diagnoses of AAD are multiple and highly heterogeneous [24]. In the present ED-based study cohort, for instance, 6.8% of patients without AAD were finally diagnosed with acute coronary syndrome, while 8.1% received a diagnosis of inflammatory disease such as pancreatitis or pneumonia, thus reflecting the wide spectrum of differential diagnosis.

In the present study, median plasma levels of MMP8 and MMP9 were respectively 2.8-fold and 2.1-fold higher in AAD than in patients receiving alternative diagnoses. The plasma levels of MMP8 and MMP9 were highly correlated, indicating that leakage of these proteins into the bloodstream relates to the same biological processes. As plasma MMP8 and MMP9 were similar in patients with uncomplicated aortic aneurysm and in patients with other unspecific diagnoses having a regular aortic size, aortic dilation *per se *does not appear as a major determinant of plasma MMP8 and MMP9 in the selected population of patients.

Based on AUC analysis, the overall performance of plasma MMP8 and MMP9 for the diagnosis of AAD is limited and inferior to that of D-dimer, the only marker of AAD currently available in clinical practice [6,7]. This is largely due a substantial variability of plasma MMP8 and MMP9 in patients without AAD, which leads to negligible specificity and positive predictive value of these markers. In particular, high circulating levels of MMP8 and MMP9 were found in patients affected by inflammatory conditions, such as pancreatitis and pneumonia. This is in line with the notion that MMP8 and MMP9 are expressed by neutrophils and with previous reports that plasma MMP9 is increased in acute pancreatitis [19,25]. Indeed, both plasma MMP8 and MMP9 slightly but significantly correlated with CRP levels in the present study population.

The present results clearly demonstrate that plasma MMP8 and MMP9 are unsuitable *per se *to diagnose AAD in patients presenting to the ED with suggestive symptoms. Instead, our data indicate that low plasma levels of MMP8 could be used to rule out AAD. For instance, rule out of AAD in the presence of MMP8 <3.6 ng/ml could avoid 7/126 urgent CT scans in the study cohort, corresponding to a 5.6% reduction in the number of urgent CT scans performed in the ED for suspected AAD. The CT scans in question were performed in four patients with uncomplicated aortic aneurysm and in three patients with other unspecific diagnoses, and did not provide any additional clinical benefit. Several studies have shown that also D-dimer has favorable rule-out properties for AAD. In our study cohort, rule out of AAD in the presence of D-dimer <0.50 µg/ml could avoid 21/103 urgent CT scans, corresponding to a 20.4% reduction in the number of urgent CT scans performed in the ED for suspected AAD. However, the latter approach would lead to one missed diagnosis of Stanford type B AAD, with presumably dramatic clinical consequences. These data indicate that, although D-dimer shows a better ROC curve than plasma MMP8 for the diagnosis of AAD, MMP8 may provide better sensitivity and lower rates of false negatives if a sufficiently low cutoff is applied.

A major finding of the present study is that combination of low plasma MMP8 and low D-dimer has the potential to safely rule out AAD. In our cohort, combination of plasma MMP8 <11.0 ng/ml and D-dimer <1.0 or <2.0 µg/ml (that is individually suboptimal cutoffs for both markers) provided a NPV of 100%. Rule out of AAD with MMP8 <11.0 ng/ml and D-dimer <1.0 or <2.0 µg/ml could avoid respectively 14/103 or 22/103 urgent CT scans, corresponding to a 13.6% or 21.4% reduction in the number of urgent CT scans for suspected AAD, without any missed diagnosis of AAD. In order to limit costs, plasma MMP8 levels might be measured only in patients with elevated D-dimer levels. In this scenario, turnaround time could be limited by applying rapid point-of-care testing of D-dimer in the ED [26].

Taken together, results from the present study indicate that the plasma levels of MMP8 may assist emergency physicians in evaluating the necessity to perform urgent secondary imaging in patients with clinical presentations suggestive of AAD. Extrapolating data from the study cohort, plasma MMP8 alone has the potential to avoid one urgent CT scan every twenty patients with a clinical suspicion of AAD, while combination of MMP8 and D-dimer could avoid as much as one urgent CT scan every five patients. In the context of a continuous increase in the rate of CT use in EDs (for example 5- and 10-fold increases in a decade for the categories of chest and abdominal pain), the benefits of such an approach will clearly depend on the respective site-specific costs, availability and turnaround times of biochemical assays and urgent secondary imaging [27].

While the struggle for a specific aortic biomarker continues, validation of robust rule-out approaches may pave our way toward a safer and more cost-effective management of suspected AAD in EDs while avoiding an exponential increase in urgent CTs.

### Limitations

A number of limitations apply to the present findings. First, data on diagnostic accuracy clearly depend on the enrolled population of patients. We applied for enrolment the diagnostic algorithm suggested by the American Heart Association [22]. However, the diagnostic performance of this algorithm is largely unknown, especially in the context of EDs [28]. Furthermore, even compliance with these guidelines does not eliminate a substantial amount of subjectivity in the clinical suspicion of AAD and thus in the decision of the caring physician to perform an urgent CT. Second, we acknowledge that sample size constitutes a significant limitation of the present study. In particular, we provide proof-of-concept that combination of D-dimer with plasma MMP8 at individually suboptimal cutoffs could provide a better rule-out profile than D-dimer alone. However, D-dimer was measured in only 103/126 patients enrolled in the present study (81.7%). Hence, conclusive demonstration of statistical superiority requires a substantial increase in patient number. Given the low incidence of AAD, multicenter studies are presumably needed for the purpose.

## Conclusions

In a prospectively enrolled cohort of patients evaluated in the ED, plasma MMP8 and MMP9 showed negligible specificity for the diagnosis of AAD. Plasma MMP8 demonstrated better diagnostic performance than plasma MMP9, but both MMP8 and MMP9 were inferior to D-dimer on ROC analysis. However, use of a low cutoff for plasma MMP8 (3.6 ng/ml) provided high sensitivity to rule out AAD and allowed a potential 5% reduction in urgent secondary imaging. Combined use of plasma MMP8 and D-dimer at individually suboptimal cutoffs provided optimal negative predictive value and allowed a potential 20% reduction in urgent secondary imaging.

## Key messages

• While plasma MMP8 and MMP9 lack specificity for AAD, low plasma MMP8 levels have satisfactory rule-out properties for AAD in the ED.

• Combination of D-dimer and plasma MMP8 could be used to avoid a significant amount of urgent secondary imaging in patients evaluated for suspected AAD in the ED.

## Abbreviations

AAD: acute aortic dissection; AUC: area under the curve; CRP: C-reactive protein; CT: computed tomography; ED: emergency department; EDTA: ethylenediaminetetraacetic acid; ELISA: enzyme-linked immunosorbent assay; IQR: interquartile range; LDH: lactate dehydrogenase; MMP8: matrix metalloproteinase-8; MMP9: matrix metalloproteinase-9; NPV: negative predictive value; ROC: receiver-operating characteristics.

## Competing interests

The authors declare that they have no competing interests.

## Authors' contributions

FG enrolled patients, acquired and analyzed the data. MLo, MLu and MM managed plasma samples and performed biochemical assays. SB, ES, EL and CM enrolled patients. GM and EH coordinated sample collection and biochemical assays. FM designed and coordinated the research, enrolled patients, analyzed and interpreted the data, wrote the manuscript. All authors have read and approved the manuscript for publication.
